# Breaking the Law: Is It Correct to Use the Converse Bergmann Rule in *Ceroglossus chilensis*? An Overview Using Geometric Morphometrics

**DOI:** 10.3390/insects15020097

**Published:** 2024-02-01

**Authors:** Hugo A. Benítez, Carlos Muñoz-Ramírez, Margarita Correa, Ian S. Acuña-Rodríguez, Amado Villalobos-Leiva, Tamara Contador, Nelson A. Velásquez, Manuel J. Suazo

**Affiliations:** 1 Centro de Investigación de Estudios Avanzados del Maule, Instituto Milenio Biodiversidad de Ecosistemas Antárticos y Subantárticos (BASE), Universidad Católica del Maule, Talca 3466706, Chile; mcorreag@ucm.cl (M.C.); avillaleiv@gmail.com (A.V.-L.); 2 Centro de Investigación en Recursos Naturales y Sustentabilidad (CIRENYS), Universidad Bernardo O’Higgins, Avenida Viel 1497, Santiago 8370993, Chile; 3 Instituto de Entomología, Universidad Metropolitana de Ciencias de la Educación, Santiago 7760197, Chile; carlos.munoz@umce.cl; 4 Centro de Ecología Integrativa (CEI), Instituto de Investigaciones Interdisciplinarias (I^3^), Universidad de Talca, Campus Lircay, Talca 3465548, Chile; ian.acuna@utalca.cl; 5 Departamento de Zoología, Facultad de Ciencias Naturales y Oceanográficas, Universidad de Concepción, Concepción 4070386, Chile; 6 Cape Horn International Center (CHIC), Puerto Williams 6350000, Chile; tamara.contador@umag.cl; 7 Millennium Nucleus of Austral Invasive Salmonids (INVASAL), Concepción 4030000, Chile; 8 Sub-Antarctic Biocultural Conservation Program, Wankara Laboratory, Universidad de Magallanes, Punta Arenas 6200000, Chile; 9 Millennium Institute Biodiversity of Antarctic and Subantarctic Ecosystems (BASE), Santiago 8330015, Chile; 10 Laboratorio de Comunicación Animal, Facultad de Ciencias Básicas, Universidad Católica del Maule, San Miguel 3605, Talca 3466706, Chile; nvelasquez@ucm.cl; 11 Instituto de Alta Investigación, Universidad de Tarapacá, Casilla 7D, Arica 1000000, Chile; suazo.mj@gmail.com

**Keywords:** converse Bergmann’s rule, centroid size, Carabidae, geometric morphometrics, body size, sexual dimorphism

## Abstract

**Simple Summary:**

Understanding the prevalence of the converse Bergmann’s rule for ectotherm animals and how often this rule is broken is of utmost importance to understand the underlying mechanisms allowing organisms to adapt to different environments and the selective pressures they face. By using the ground beetle *Ceroglossus chilensis* as a biological model, we provide a practical example of testing the converse Bergmann rule in an ectotherm with a narrow geographical distribution in Chile.

**Abstract:**

The converse Bergmann’s rule is a pattern of body size variation observed in many ectothermic organisms that contradicts the classic Bergmann’s rule and suggests that individuals inhabiting warmer climates tend to exhibit larger body sizes compared to those inhabiting colder environments. Due to the thermoregulatory nature of Bergmann’s rule, its application among ectotherms might prove to be more complicated, given that these organisms obtain heat by absorbing it from their habitat. The existence of this inverse pattern therefore challenges the prevailing notion that larger body size is universally advantageous in colder climates. *Ceroglossus chilensis* is a native Chilean beetle that has the largest latitudinal range of any species in the genus, from 34.3° S to 47.8° S. Within Chile, it continuously inhabits regions extending from Maule to Aysen, thriving on both native and non-native forest species. Beyond their remarkable color variation, populations of *C. chilensis* show minimal morphological disparity, noticeable only through advanced morphological techniques (geometric morphometrics). Based on both (1) the “temperature–size rule”, which suggests that body size decreases with increasing temperature, and (2) the reduced resource availability in high-latitude environments that may lead to smaller body sizes, we predict that *C. chilensis* populations will follow the converse Bergmann’s rule. Our results show a clear converse pattern to the normal Bergmann rule, where smaller centroid sizes were found to be measured in the specimens inhabiting the southern areas of Chile. Understanding the prevalence of the converse Bergmann’s rule for ectotherm animals and how often this rule is broken is of utmost importance to understand the underlying mechanisms allowing organisms to adapt to different environments and the selective pressures they face.

## 1. Introduction

One of the most used ecogeographical rules is the one Carl Bergmann published in 1847. Even though the exact translation of this rule initially published in German has been a matter of controversy [1], there is a consensus that Bergmann’s rule predicts that the body size of living organisms increases as temperature decreases [2,3,4]. This size growth is related to a thermoregulatory phenomenon in endotherm organisms; as the ratio of volume versus surface area increases in animals, they retain heat better, and this can be explained by the square–cube law, which predicts that volume will increase faster than the surface area [3,5]. On the contrary, the converse Bergmann’s rule is a pattern of body size variation observed in many ectothermic organisms that contradicts the classic Bergmann’s rule and suggests that individuals living in warmer environments tend to be larger than those living in colder environments [3,6,7].

Some studies have explained Bergmann-type clines by other features, different from thermoregulation, such as dispersal, resource, habitat and/or genetics [1,5,8,9,10,11]. This rule has been demonstrated for many organisms, for example, Meiri and Dayan [12], with a focus on endothermic organisms, analyzed 94 species of birds and 149 species of mammals and found that 72% and 65% of them, respectively, follow Bergmann’s rule. In this way, the authors conclude that this rule can be a valid ecological generalization for these two groups of endotherms. However, when studying the application of Bergmann’s rule in major ectotherm groups, contrasting responses can be observed [7,13,14]. While Anurans increase their body size with latitude, lizards and snakes (squamates) reverse (or converse) this rule by decreasing their size as latitude increases [15,16].

Indeed, as Bergmann’s rule involves a thermoregulatory process, its generalization in ectotherms may be more complicated as they obtain heat by absorbing it from the environment [13,17]. As volume increases, organisms require more heat to alter their body temperature. This adaptation is likely beneficial in extreme climates. Conversely, a larger surface area combined with reduced volume allows for quicker heat absorption, but also results in faster heat loss, making it a strategy potentially favored in more stable climates. An explanation is that ectotherms will grow slowly in a colder climate but will finally reach a more significant size due to increased cell size [18,19]. The question of whether ectotherms adhere to a converse Bergmann’s rule has been contentious [7].

In understanding the prevalence of the converse Bergmann’s rule for ectotherm animals and how often this rule is broken, it is of utmost importance to understand the underlying mechanisms allowing organisms to adapt to different environments and the selective pressures they face.

However, recent research offers empirical evidence of an inverse relationship between body size and latitude. This challenges conventional understanding and calls for a closer examination of the underlying mechanisms [6,8,20]. For example, in high-latitude regions like the Arctic, sub-Arctic and sub-Antarctic ecosystems, smaller-bodied individuals (groups) have been documented, in contrast to those found at lower latitudes, with larger-bodied groups [21]. The existence of this converse pattern challenges the prevailing notion that larger body size is universally advantageous in colder climates.

Nevertheless, since current evidence suggests that different ectotherm taxa could express different clinal patterns in regard to Bergman’s rule, a detailed exploration of this distributional pattern is needed [7,13]. For example, insects are considered one of the largest ectothermic groups in the world, and this size–temperature-dependent rule is “followed” only by some species [3]. Shelomi [3] states the importance of well-designed and continuous intraspecific studies as patterns regarding this rule can vary substantially even between closely related species [22]. Even though most species of insect groups are more likely to follow the converse Bergmann’s rule (such as Coleoptera), some groups exactly follow Bergmann’s rule (Diptera) or show no significant trends, like Plecoptera [3], water beetles [23] or even Ephemeroptera [14]. Interestingly, when the phylogenetic inertia associated with an insect group was controlled through comparative phylogenetic analyses, an insect group followed the converse Bergmann’s rule; see the case of the bumblebee in [24].

**Figure 1:**
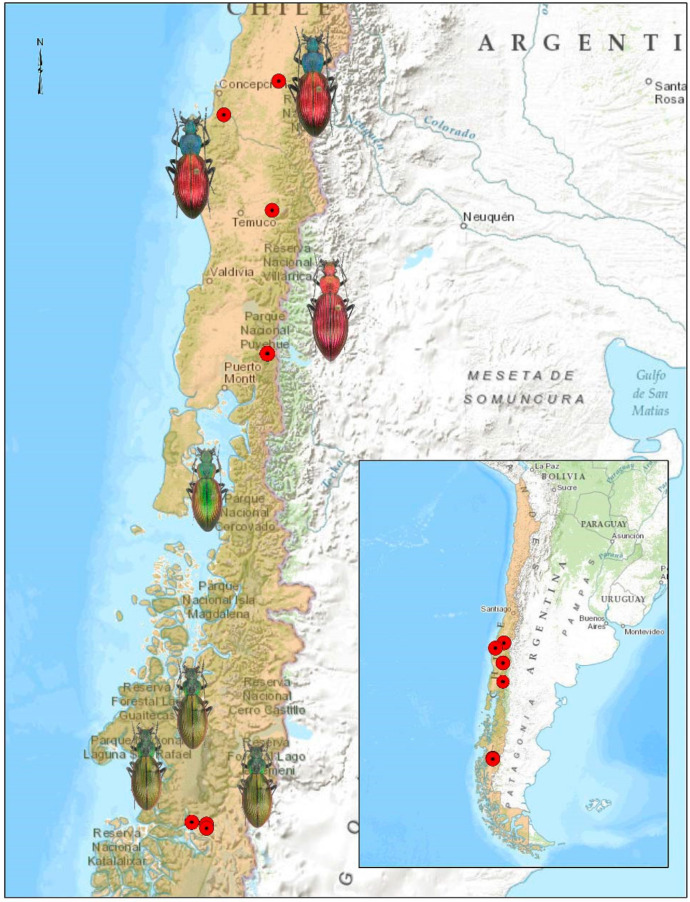
Distribution of *Ceroglossus chilensis* across their whole latitudinal cline in Chile; points represent north (CC, PC), center (MZ, PM) and south (L1, L2, L3) localities from their distribution.

The genus *Ceroglossus* (Coleoptera: Carabidae) encompasses a group of colorful ground beetles endemic to temperate forests of southern South America [25,26]. The genus comprises eight described species: *C. chilensis* (Eschscholtz), *C. darwini* (Hope), *C. speciosus* Gerstaecker, *C. magellanicus* Géhin, *C. buqueti* (Laporte), *C. suturalis* (Fabricius), *C. ochsenii* (Germain) and *C. guerini* (Germain), although it may harbor higher taxonomic diversity [27]. Most of the species exhibit a striking pattern of sympatric color convergence hypothesized as a product of Müllerian mimicry [28]. *Ceroglossus chilensis*, also known as the magnificent Chilean beetle, has the largest distribution range among all these species (from −34.3° to −47.8°, Figure 1). In Chile, it has a continuous distribution from the Maule Region to the Aysen Region and can be found in native and exotic forest species [29,30]. Aside from their high color diversity, populations of *C. chilensis* exhibit little morphological differences that are only detected when using advanced morphological tools [25,31,32]. In addition, the species presents sex and size dimorphism [25,33]. Benitez et al. [31] found for *Ceroglossus chilensis* that disparities in morphology and variations among sampling sites in the southern population in Chile stem from differences in shape rather than size; their results suggest that size variations among populations are inevitably shaped by environmental influences. Nevertheless, the historical impact of anthropogenic activities has introduced disturbances in the Aysén Region, contributing to a profoundly heterogeneous vegetation landscape.

Based on both (1) the “temperature–size rule”, which suggests that body size decreases with increasing temperature, and (2) the reduced resource availability in high-latitude environments that may lead to smaller body sizes, we predict that *C. chilensis* populations will follow the converse Bergmann’s rule.

## 2. Materials and Methods

*Ceroglossus chilensis* description: Adults exhibit an elongated form and considerable variability in body coloration; the head, thorax and elytra display iridescent shades of black, green and blue. Ventrally, they are black, as are the legs, maxillary palps and labial palps. The head and pronotum are blue and covered with fine punctuations. The elytra, in general, are very shiny, convex and elongated, with a dark red coloration. Sexual dimorphism is subtly apparent and can be observed in variations in the shape of the pronotum, elytra and abdomen, resulting from intrasexual competition. Males exhibit keels (carinae) on the antennal segments 6, 7 and 8. The tarsi of the first pair of legs are widened, and the apex of the elytra is rounded [34]. Dorsally, the elytra are sculpted and have pronounced humeral angles. Metathoracic wings are absent [34,35,36]. In females, the pronotum is wider and longer compared to males, with a small longitudinal line along the midline; the abdomen is larger, interpreted as an adaptation for egg production, and the elytra are more pointed at the apex [29].

Generally, for *Ceroglossus* species, the developmental cycle lasts approximately three months [29]. For oviposition, females construct galleries in the soil where they deposit eggs, numbering from 10 to 15. From laying, it takes an average of two weeks for the eggs to hatch. The development times of different larval stages vary; for example, the first larval stage lasts two weeks, the second stage lasts three weeks and the third stage lasts two weeks. Larvae always molt on the surface, hidden under pieces of bark [29]. Subsequently, the larva buries itself 10 cm in the soil and constructs a pupal chamber within which it remains immobile. This last stage lasts for about two weeks, during which certain segments and body parts are pigmented (tibiae, eyes, mandibles). At the end of metamorphosis, the fully decolorized imago emerges, and the chromatogenesis period lasts about 24 h. Adults are preferably found in January, February and March [34,35,36].

Sampling: Pitfall traps were placed in isolated geographic areas across the whole latitudinal distribution of *Ceroglossus chilensis* in Chile.

Two localities were selected in the northern part of their distribution: (1) the coastal mountain range (CC, Santa Juana, 37.1750° S, 72.9457° W) and (2) the Andes foothill (PC, Coihueco 36.62611° S 71.83444° W). Two localities were selected in the center, namely (1) Manzanares (MZ, 38.4060° S, 71.5961° W) and (2) Puyehue (PM, 40.6694° S, 72.1720° W), while three were selected in the southern distribution limit: L1, L2 and L3 (Aysen; 47.79139° S, 73.56778° W). Twelve traps were installed separated approximately 5 m from each other, for 3 days and 3 nights (Figure 1).

**Figure 2:**
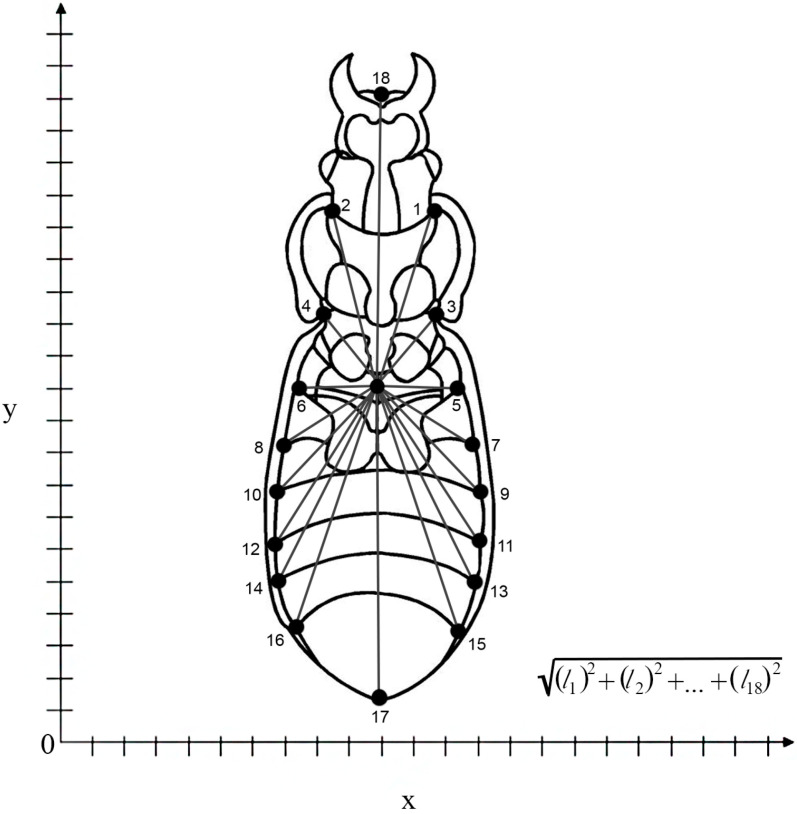
Schematic representation of the landmarking procedure in *Ceroglossus chilensis*; the center point represents the gravity center of the distance between every landmark used to calculate the centroid size.

In the geometric morphometric analysis, complete variation in shape was considered, and this analysis was performed using a ventral view of males and females with an Olympus X-715 digital camera (Olympus, Tokyo, Japan). Following the methodology described in [31], eighteen landmarks (LMs, anatomical homologous points) were digitized on every picture using the software TpsDig v2.31 [37] (Figure 2). Once the 2D x-y coordinates were obtained for all landmarks, the shape information was extracted using a Procrustes fit. This procedure, also called Procrustes superimposition, is a procedure that removes the information of size, position and orientation to standardize each specimen according to centroid size [38,39]. A Procrustes ANOVA in the software MorphoJ 1.07a was first calculated to compare a first set of landmarking processes with a second to determine if there was any measurement error in the digitalization procedure [40,41]. After that, using the revised dataset, a covariance matrix of shape individuals was performed to calculate all the multivariate analyses of shape. A principal component analysis (PCA) was performed to simulate the shape space, and the first three components were quantified. The proxy of geometric size was analyzed using the centroid size, which represents the center of the landmark configuration [42] (Figure 2).

The centroid size was computed as the average of the 2D coordinates of all landmarks and was calculated as the square root of the summed squared distances. This proxy is a single value that provides an approximation of overall size considering shape differences [38,42]. A generalized linear mixed model (GLMM) was used to evaluate the overall effect of both sex and zone of location (north, center and south) on the centroid size of the sampled individuals. This method allows the inclusion of fixed (sex and locality) and random (individuals within populations) factors in model structure, while managing to fit non-Gaussian response distributions [43]. Accordingly, after exploring the residuals of the response variable under different density distribution scenarios, we used the Gamma distribution (log-link), which has been observed to better capture the variability of right-skewed response distributions [44]. In addition, to evaluate the population as a factor within each zone, independent GLMM models were fitted for the south, center and north datasets including the population as a fixed factor. In addition, to evaluate the effect of sex in the body morphometrics in each population, independent *t*-tests were performed for each population dataset. The R language and environment v.4.2.0 (R-CoreTeam 2022) was used for all the analyses; GLMM analysis was performed through the “glmer” function from the *lme4* R-package [45].

Finally, to examine the differences in shape between localities, a Procrustes ANOVA and a canonical variate analysis were performed between sex and localities. In addition, multivariate regression was performed in order to quantify allometric differences between shape and size, using the centroid size as an independent variable and after running a permutation test with 10,000 rounds using the software Morphoj 1.07a [46].

## 3. Results

**Figure 3:**
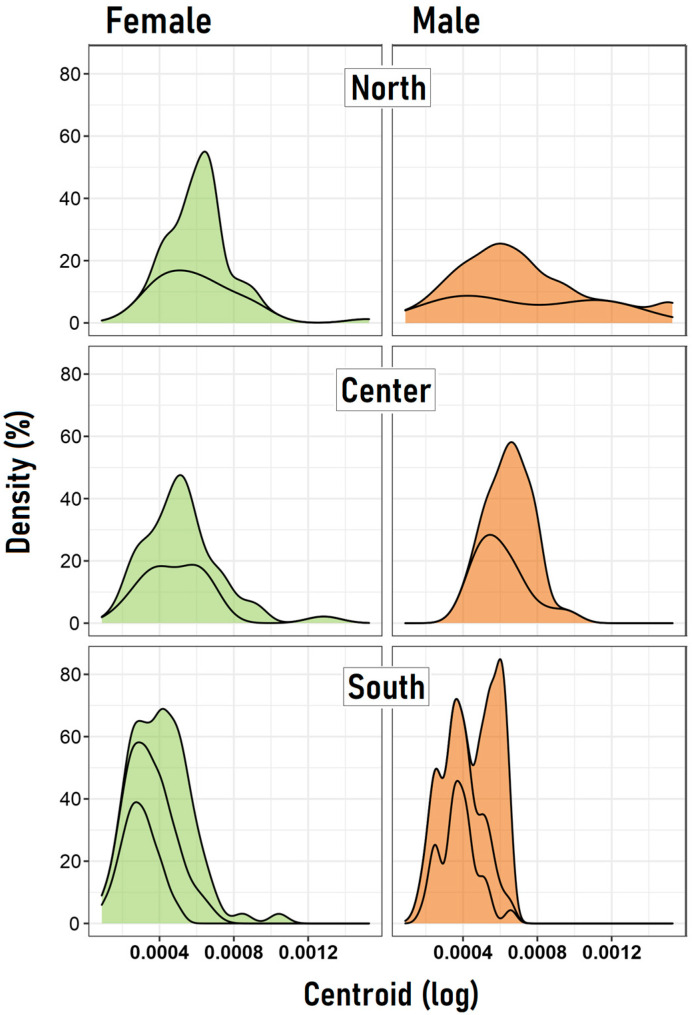
Density (%) distribution of the log-centroid data by sex, zone and population.

First, geometric morphometric results indicate that using the Procrustes ANOVA to calculate the digitizing error shows that the mean square for individual variation exceeded the measurement error (0.000106 < 0.0000362), which means ME is small enough to proceed with further analyses. The first three principal components (PCs) accumulated 58.9% of the shape variation (PC1 = 26.2%, PC2 = 18.9% and PC3 = 13.6%). Using the centroid size, the overall GLMM model failed to detect differences in the density distributions of centroid values between females and males across zones but did find a significant effect of the zone independent of the sex (Table 1). Between the north and center zones, no differences were observed, probably due to their large intra-population variability. However, both the north and center zones significantly differ from the south, and this was observed in both females and males (Figure 3).

In addition, the zone-specific GLMM models point to a similar direction; differences between the studied factors (sex and population) increase in relevance towards southern latitudes (Table 1).

**Figure 4:**
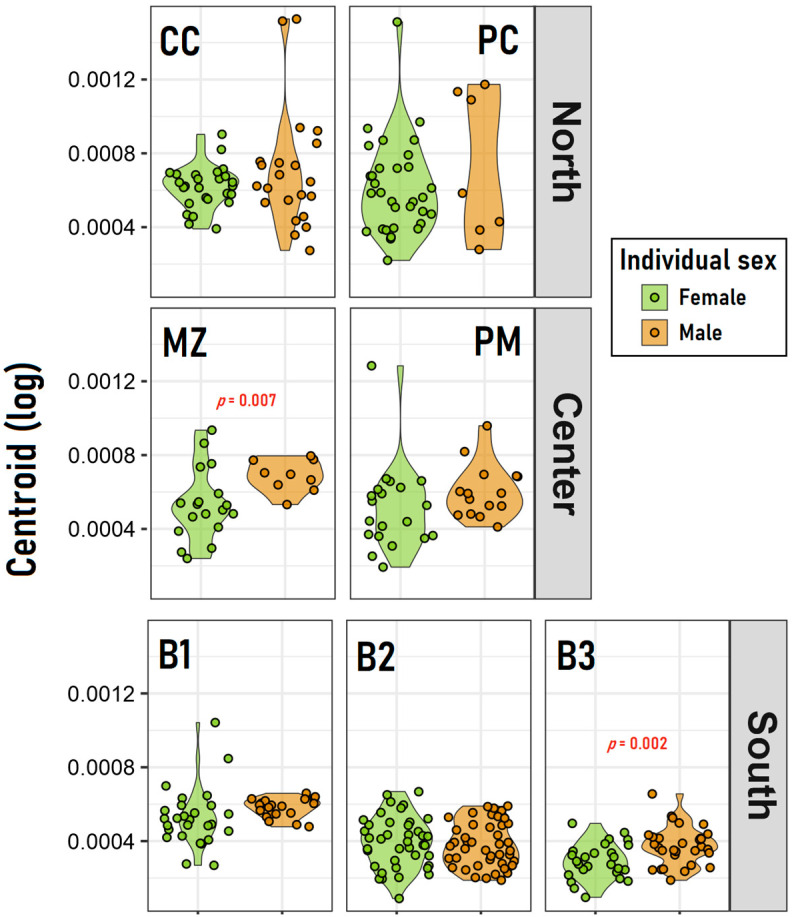
Comparison of the log-centroid values between sexes (female–male) by populations within each of the three geographic zones (North, Center and South). The probability (*p*) values are indicated only for those populations for which the *t*-test was significant (*p* < 0.05).

In the north, neither the sex nor the population resulted as significant in determining the distribution of the centroid values; by contrast, both factors appeared to influence this distribution in the south zone. Also, in the center zone, sex was a significant factor in the model, nevertheless, in both the center and south zones, the overall differences between females and males seem to be driven by some populations more than others (Figure 4). This was corroborated by the independent *t*-tests realized between both sexes within each population (Table 2), which clearly denoted that females and males significantly differ in their log-centroid values only in the MZ (center) and the B3 (south) populations.

**Figure 5:**
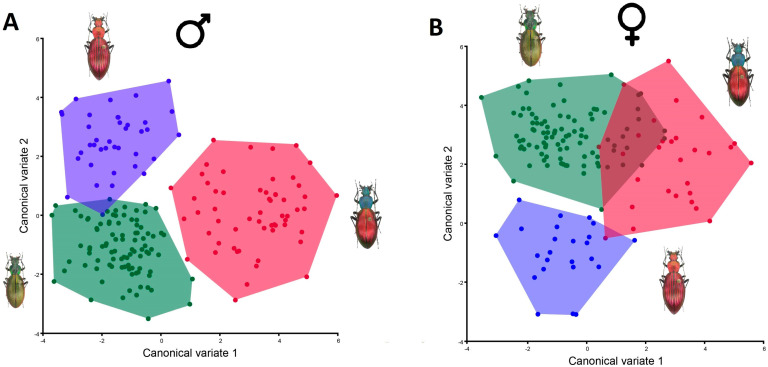
Canonical variate analyses of the body shape in *Ceroglossus chilensis* geographical distribution and between sexes. Colors represent the north (red), center (blue) and south (green).

The canonical variate analysis showed three clearly identified groups from the north, center and south localities (Figure 5). This shape variation was principally determined by the vector movement of landmark 18 for females between north and south, which also is related to the elongation of the body shape. A widening of the morphology product of the contraction of the pairs of landmarks 13–14 and 15–16 also was noticed. For males also, landmark 18 noticeably changes between north and south; nevertheless, there is less variation between individuals of the center of the distribution with a left contraction of the pair of landmarks 5–6, where the abdomen begins, and a bit of elongation of the end of the thorax at landmarks 3 and 4. All these modifications in shape were statistically significant by sex and locality (ANOVA by sex: *F*: 53.28, *p*: 0.004; ANOVA by locality: F: 55.69, *p*: <0.001).

## 4. Discussion

Since the article about Bergmann’s rule was published in 1847, there has been a continuous debate regarding its applicability in ectothermic organisms such as insects and vertebrates. This is primarily due to the thermoregulatory mechanisms that do not consistently explain patterns observed in these study models. Contradictory results have been observed among studies, further fueling the discussion [10,11,23,47].

Several authors mention that the variability of sizes according to latitude can respond to different factors, which are different from each other. These different factors, originating from different climatic causes, when acting together, produce many intermediate patterns, which in turn are determined by the taxon in the studio or the locality of origin [3,10,11,23,48].

In the case of *C. chilensis*, we analyzed and questioned this pattern in terms of the sexual traits, to evaluate if there are differences between size and sexes along the latitudinal gradient studied. Nevertheless, there is no generalized pattern of sexual dimorphism, except for the MZ locality in the center of the sampled area. This result may indicate a more plastic response to the selective pressures of the species that are not necessarily linked to the latitude or the temperature conditions. Similarly, in terms of the body size pattern, the results indicate the presence of a negative correlation between size and latitude, showing significantly smaller individuals towards higher latitudes. It is also important to point out that more than the body sizes themselves, significant differences are observed in the frequencies of the different size categories. It is observed that although the mean size of individuals collected in the north of the distribution is similar to the mean size of individuals collected in the center, the northern zone shows a greater amplitude of variation in body size, while in the central and southern zones, the amplitude of variation in body size is much more reduced. These results could indicate an effect of the environmental variability associated (temperature of microhabitats) with the different localities. Since the northern zone is less climatically variable, there would be greater climatic opportunities for the appearance of different generations, representing a multivoltine pattern [8,49,50,51]. If this pattern is associated with favorable climatic conditions of shorter duration between unfavorable conditions, it could indicate a shorter duration of the larval stages, which would produce smaller adults, and likewise, favorable periods of longer duration would produce larger adults [51,52,53,54,55]. This pattern is only observable in males of *C. chilensis*, which could be a sign of a high-competition scenario among males from the northern zone [33], because females have less variable body sizes. A similar pattern has been observed in the body sizes of various species of arthropods subjected to environmental variability, where a large variation in body sizes associated with the duration of the season or the temporal variation of climatic and environmental events is observed [31,56,57,58].

As for individuals from the central and southern zones, a similar pattern is observed in both males and females, where the tendency is for smaller body sizes at high latitudes, with lower variability. These results are concordant with the ones obtained by Baranovská and Knapp [6] in beetle species, where a converse Bergmann’s clinal pattern was found across an altitudinal gradient in four of the eight species studied, while the body sizes of the other four species showed no pattern.

Shelomi [3] indicates that the majority of the studies that examine the relationship between body size and latitude/altitude in arthropods show that there is no relationship, especially when analyzed over wide geographical ranges or in interspecies comparisons, but also that patterns emerge mainly in studies of restricted geographical ranges or between populations of the same species. This could give us clues that, if the mechanisms underlying the processes of body variation in arthropods are not completely clear [3,10,11,23,59], variation could be a response to ecological conditions associated with the altitudinal gradient, without necessarily being thermoregulatory in nature [1,2,47,48], and may simultaneously respond to different causes. An example of this has been observed by Romero et al. [60] in an experiment carried out all over the world, where they observed the size variation of arthropods as a function of the use of microclimatic shelters, such as the leaf curling produced by some arthropods. Thus, they observed that body size is inversely proportional to temperature and aridity, as it increases with precipitation, without observing an effect of latitude or altitude. Alternatively, Gérard et al. [10] show a positive relationship between body size and latitude in different species of bees, but they suggest that this differentiation seems to be associated with social behavior, flight behavior and the nesting strategy of the different species, as also reported in beetles that follow the converse Bergmann’s rule. Sanzana et al. [20] reported another species following the converse Bergmann’s rule. The butterfly species *Auca coctei* presents a negative relationship between the size of the wings of the females and latitude, and the same pattern is not reported in the males of the same species, which could give evidence of sexual selective pressures not necessarily related to temperature. Similarly, Pallares et al. [23] found an unclear pattern in the latitudinal size variation between different lineages of water beetles of the Dytiscidae family, observing an effect related to the habitat preference at the interspecific level. In addition, a remarkable case is that of the dragonfly species *Nannophya koreana* (Odonata: Libellulidae), where it was observed that there is a negative relationship between body size and temperature on a scale of 120 km, and it was emphasized that this variation is related to the temperature of the water where the larvae of the species develop [61].

It should be noted that although our results show that *C. chilensis* presents a pattern apparently concordant with the converse Bergmann’s rule, these results should be treated with caution, since Bergmann’s rule is a pattern associated with thermoregulation, and its applicability in ectotherms has been widely questioned [7]. On the other hand, these results could be related to unknown ecological factors associated with an environmental gradient, without necessarily having a simple causal relationship. This work provides new evidence for a pattern of body size variation in a latitudinal context, suggesting that *C. chilensis* is a good model species for studying the mechanisms underlying size variation in an ecological context.

## Figures and Tables

**Table 1:** GLMM model parameters and statistics for the complete dataset (All) and by geographic zones (North, Center and South). In each case, the compared factor levels are in parentheses. Values of *p* indicate statistical significance (*p* < 0.05) in explaining the respective model variance.

Trait	Factor	*b*	SE	*t*	*p*
All	Intercept	−7.516	0.140	−53.54	<0.0001
	Sex (female–male)	0.098	0.111	0.87	0.3804
	Zone (C–N)	0.137	0.175	0.78	0.4320
	Zone (C–S)	−0.322	0.155	−2.07	0.0378
	Zone (N–S)	−0.460	0.140	−3.28	0.0010
North	Intercept	−7.414	0.065	−113.61	<0.0001
	Sex (female–male)	0.077	0.085	0.90	0.3660
	Population (PC–CC)	−0.055	0.081	−0.68	0.4960
Center	Intercept	−7.592	0.068	−110.53	<0.0001
	Sex (female–male)	0.270	0.085	3.17	0.0015
	Population (PM–MZ)	−0.081	0.085	−0.96	0.3290
South	Intercept	−7.575	0.051	−145.80	<0.0001
	Sex (female–male)	0.094	0.048	1.96	0.049
	Population (B2–B1)	−0.384	0.059	−6.42	<0.0001
	Population (B3–B1)	−0.529	0.064	−8.14	<0.0001
	Population (B3–B2)	0.140	0.056	2.48	0.0131

**Table 2:** Independent *t*-test between female and male centroid values (log) within each sampled population. CI: confidence interval (95%, low or high), *t*: *t*-statistic, *d.f.*: degrees of freedom, *p*: probability value. *p*-values show those populations for which the difference between the sex group means was statistically different from zero (*p* < 0.05).

Zone	Population	Mean Female	Mean Male	Mean Diff	CI-Low	CI-High	*t*	*d.f.*	*p*
North	CC	0.000616	0.000701	−8.55 × 10^−5^	−2.3 × 10^−4^	6.1 × 10^−5^	−1.199	25.62	0.2411
	PC	0.000612	0.000725	−1.13 × 10^−4^	−4.7 × 10^−4^	2.5 × 10^−4^	−0.727	7.12	0.4904
Center	MZ	0.000531	0.000688	−1.56 × 10^−4^	−2.6 × 10^−4^	−4.6 × 10^−5^	−2.922	24.91	0.0072
	PM	0.000512	0.000605	−9.25 × 10^−5^	−2.2 × 10^−4^	3.8 × 10^−5^	−1.444	32.15	0.1581
South	B3	0.000291	0.000375	−8.34 × 10^−5^	−1.3 × 10^−4^	−3.0 × 10^−5^	−3.137	53.72	0.0027
	B2	0.000395	0.000382	1.34 × 10^−5^	−4.3 × 10^−5^	7.0 × 10^−5^	0.471	77.81	0.6389
	B1	0.000527	0.000579	−5.18 × 10^−5^	−1.1 × 10^−4^	1.4 × 10^−5^	−1.587	32.95	0.1219

## Data Availability

Data of this study can be requested by mailing the corresponding author.
